# *Plasmodium falciparum* malaria parasite *var* gene expression is modified by host antibodies: longitudinal evidence from controlled infections of Kenyan adults with varying natural exposure

**DOI:** 10.1186/s12879-017-2686-0

**Published:** 2017-08-23

**Authors:** Abdirahman I. Abdi, Susanne H. Hodgson, Michelle K. Muthui, Cheryl A. Kivisi, Gathoni Kamuyu, Domtila Kimani, Stephen L. Hoffman, Elizabeth Juma, Bernhards Ogutu, Simon J. Draper, Faith Osier, Philip Bejon, Kevin Marsh, Peter C. Bull

**Affiliations:** 1 KEMRI-Wellcome Trust Research Programme, CGMRC, P.O. Box 230-80108, Kilifi County, Kenya; 2 Pwani University, P. O. Box 195-80108, Kilifi, Kenya; 3 The Jenner Institute, University of Oxford, Oxford, UK; 4 Centre for Clinical Research, Kenya Medical Research Institute, Nairobi, Kenya; 5 Centre for Research in Therapeutic Sciences, Strathmore University, Nairobi, Kenya; 6 Sanaria Inc., Rockville, MD USA; 7 Department of Pathology, University of Cambridge, 17 Tennis Court Road, Cambridge, CB2 1QP UK

**Keywords:** PfEMP1, Antibodies, *P. falciparum*, Immunity, Controlled human malaria infection (CHMI), Sporozoite

## Abstract

**Background:**

The PfEMP1 family of *Plasmodium falciparum* antigens play a key role in pathogenesis of severe malaria through their insertion into the surface of parasite infected erythrocytes, and adhesion to host cells. Previous studies have suggested that parasites expressing PfEMP1 subclasses group A and DC8, associated with severe malaria, may have a growth advantage in immunologically naïve individuals. However, this idea has not been tested in longitudinal studies.

**Methods:**

Here we assessed expression of the *var* genes encoding PfEMP1, in parasites sampled from volunteers with varying prior exposure to malaria, following experimental infection by sporozoites (PfSPZ). Using qPCR, we tested for associations between the expression of various *var* subgroups in surviving parasite populations from each volunteer and 1) the levels of participants’ antibodies to infected erythrocytes before challenge infection and 2) the apparent in vivo parasite multiplication rate.

**Results:**

We show that 1) expression of *var* genes encoding for group A and DC8-like PfEMP1 were associated with low levels of antibodies to infected erythrocytes (αIE) before challenge, and 2) expression of a DC8-like CIDRα1.1 domain was associated with higher apparent parasite multiplication rate in a manner that was independent of levels of prior antibodies to infected erythrocytes.

**Conclusions:**

This study provides insight into the role of antibodies to infected erythrocytes surface antigens in the development of naturally acquired immunity and may help explain why specific PfEMP1 variants may be associated with severe malaria.

**Trial registration:**

Pan African Clinical Trial Registry: PACTR201211000433272. Date of registration: 10th October 2012.

## Background

When *Plasmodium falciparum* malaria parasites infect erythrocytes, they insert proteins into the erythrocyte surface that alter the properties of the infected erythrocyte surface. A large component of these inserted proteins is *P. falciparum* erythrocyte membrane protein1 (PfEMP1) [1]. This family of parasite proteins play a key role in the pathology of severe malaria by mediating the cytoadhesion of infected erythrocytes (IE) to endothelial cells and other uninfected erythrocytes leading to IE sequestration in the microvasculature. This is thought to promote parasite survival by avoiding clearance by the spleen (reviewed in [2, 3]). Cytoadhesion is mediated by two broad categories of adhesive domains called DBL and CIDR domains, arranged in highly diverse combinations like beads on a string [4]. Because of their exposure on the surface of IE for long periods during blood-stage infection, PfEMP1 are key targets of naturally acquired immunity [5]. To evade host antibodies, *P. falciparum* switches between around 60 members of a diverse genomic repertoire of *var* genes, using an epigenetic mechanism that ensures only one PfEMP1 antigen is expressed at any one time by each parasite [6]. The *var* genes within each parasite genome expressed during childhood malaria can be broadly classified through their upstream promotor types: every parasite genome has a small number of *var* genes with ups A and ups C promotors, with the majority having ups B promotors [7].

The usefulness of PfEMP1 as vaccine targets is potentially limited by their extreme molecular diversity [8, 9]. However, children growing up in malaria endemic areas do develop antibodies to a broad range of PfEMP1 variants and despite their molecular diversity, expression of restricted subclasses of relatively conserved PfEMP1 variants with representative *var* genes in every parasite genome, have been found to be associated with severe malaria [10–18]. The most important defined subsets of PfEMP1 in this regard are those with an ups A promotor, called “group A” and those containing CIDRα1 domains predicted to bind to endothelial protein C receptor (EPCR) [19]. Though CIDRα1 domains have been identified within *var* genes with various adhesive domain architectures, they are frequently found in the context of commonly occurring combinations of cytoadhesive domains called “domain cassettes (DC)” [7]. Two examples of CIDRα1-containing DC reported to be associated with severe malaria [16] are DC13 (defined as: DBLα1.7, CIDRα1.4) and DC8 (defined as: DBLα2, CIDRα1.1, DBLβ12, DBLγ4/6). DC13 forms a subset of group A PfEMP1, while DC8, as strictly defined, forms a subset of *var* genes with ups B promotors (group B) [7]. In 3D7, the DBLα1.7, and CIDRα1.4 domains that make up DC13 are present within a single gene PF11_0521. DC8 as strictly defined, is absent from 3D7. However, PF3D7_0600200 (PFF0010w) and PF3D7_0800300 (PF08_0140) are DC8-like in every respect apart from having CIDRα1.8 and CIDRα1.6 domains respectively in place of CIDRα1.1. PF3D7_0400400(PFD0020c), is DC8-like in every respect apart from having a DBLα1.2 domain in the place of DBLα2, making it a group A *var*.

The fitness advantages provided by these *var* gene subsets associated with severe malaria are not known. It is possible that adhesion to EPCR by CIDRα1 increases the ability of parasites to bind to diverse endothelial cells, hence lowering the rate of parasite clearance in the spleen [20]. Alternatively, because group A and DC8 PfEMP1 tend to be relatively long genes they may have more options for cytoadhesion, again allowing them an enhanced ability to avoid passage through the spleen and sustain infections [16, 21, 22]. Because these molecules tend to be more conserved, parasites expressing these variants tend to be recognised by children who have a well-developed repertoire of anti-PfEMP1 antibodies [23, 24]. Therefore, naturally acquired antibodies against these restricted subclasses of PfEMP1 variants have been proposed to confer protection against severe disease [25, 26].

This hypothetical trade-off between cytoadhesion and immune escape leading to the evolution of a subclass of PfEMP1 variants with a growth advantage in immunologically naïve hosts is an attractive idea; it potentially helps explain the development of immunity to severe malaria in the first few years of life despite immense overall molecular diversity in the PfEMP1 family of proteins. Several pieces of evidence indirectly support the idea. Firstly, recombinant proteins made from Group A PfEMP1 and DC8 tend to be more commonly recognized than other group B and C PfEMP1, suggesting they are antigenically more conserved [26–28]. Secondly, expression levels of group A-like PfEMP1 were associated with both severe malaria and absence of antibodies at the time of disease and young host age [10]. However, direct evidence is still needed to support 1) differential survival of this subclass of PfEMP1 variants in the face of different levels of pre-existing naturally acquired immunity and 2) an intrinsic growth advantage over other PfEMP1 types in the absence of antibody pressure, as opposed to a purely passive relation with prior exposure that is driven by their relative conservation [10, 11].

Here, using a controlled human malaria infection (CHMI) study of twenty-eight Kenyan adults infected by intramuscular administration of aseptic, purified, cryopreserved NF54 (the parent line of 3D7) *P. falciparum* sporozoites, (Sanaria® PfSPZ Challenge) [29], we show that pre-existing antibodies to IE (αIE) were associated with reduced group A and DC8-like *var* gene expression. One of three sets of primers (dc13, dc8-1 and dc8-2) used to amplify CIDRα1 domains, dc8-1, amplified a *var* signal associated with apparent in vivo parasite multiplication rate (PMR) after adjustment for prior levels IE antibodies.

## Methods

### CHMI study design

An open label, randomized, CHMI pilot study using PfSPZ Challenge (aseptic, purified, cryopreserved, infectious NF54 *P. falciparum* sporozoites (Sanaria® PfSPZ [30]) was undertaken in Nairobi, Kenya [29]. Briefly, increasing doses of PfSPZ Challenge were administered intramuscularly to twenty-eight volunteers with varying degrees of prior exposure to malaria [29]. Subjects were grouped into those with minimal prior exposure to *P. falciparum* (MinExp) (*n* = 14) and those with definite prior exposure to *P. falciparum* (DefExp) (*n* = 14) determined by antibody levels to recombinant merozoite surface protein-2 (MSP-2) and whole schizont extract measured prior to CHMI [29]. The rationale for using these antigens to screen for previous exposure was based on previous published data to support their association with prior exposure [29, 31–33]. All subjects were successfully infected with malaria parasites [29]. A blood sample was collected for parasite *var* gene expression analysis upon blood-film positivity or at day 21 (C + 21) post-CHMI in those undiagnosed by this time-point. This blood sample was processed as previously described [10, 11], with 100 μl of RBCs, depleted of white blood cells, re-suspended in TRIzol and stored at −80 °C until use. All subjects were treated with a curative course of atovaquone/proguanil either when blood-film positive or at C + 21.

### Parasite DNA extraction and 18S ribosomal RNA gene PCR

qPCR for *P. falciparum* 18S ribosomal RNA (18S rRNA) gene was performed on samples collected once or twice daily as previously described [34]. These data were used to calculate each subject’s PMR using simple linear regression. PMR is the fold change in number of parasites in the blood over one lifecycle (48 h). *P. falciparum* schizonts usually contain approximately 20 merozoites [35]. If these successfully invade a different red blood cell, the PMR would be 20. In CHMI studies including individuals with no naturally acquired immunity to malaria, PMR has been reported to range between 10 and 15 [34, 36, 37].

### Parasite RNA extraction and cDNA synthesis

Parasite RNA extraction and cDNA synthesis was performed as previously described [10, 11].

### *Var* transcript quantification using quantitative PCR

Primers (Table 1) previously used to quantify expression of broad classes of *var* genes in qPCR [16, 38, 39] were applied as described in [40, 41]. Two housekeeping genes, Seryl tRNA synthetase and Fructose bisphosphate aldolase [16, 39] were used as reference genes for relative quantification of the expressed *var* genes. Primers targeting the individual *var* genes in 3D7 [39] were not used as the amount of parasite RNA material available for analysis was extremely limited given the low-density parasitaemia present in subjects at diagnosis. The primers used included some designed based on 3D7 genome [38] to target group A, B and C *var* genes (Table 1). These included two group A-targeting primers A2 and A3 renamed here as gpA3 and gpA4 (Table 1) that target the exon2 region of group A genes [38]. A third broadly specific group A primer gpA1 (originally named “Dbla1 not var3”) has been shown to amplify all DBLα domains from group A 3D7 *var* genes ([16] and (Table 1).

**Table 1:** The list of primers used in this study and their predicted targets

Original primer name	Name given	Ref.	Targets in 3D7 [16]	Predicted NF54 target genes [16]
dbla_not_var3	gpA1	[16]	All group A *var* (DBLα1.2, DBLα1.3, DBLα1.4, DBLα1.5, DBLα1.6, DBLα1.7)	**PFD0020c, PFA0015c, MAL6P1.314, PFI1820w, PFD1235w, PFE1640w, PF11_0008, PF08_0141, PF11_0521, PF13_0003**
A2	gpA3	[38]	Exon2 of group A *var*	
A3	gpA4	[38]	Exon2 of group A *var*	
cidra1.4	dc13	[16]	DC13 group A *var* (CIDRα1.4)	**PF11_0521**
cidra1.1	dc8-1	[16]	One group A, DC8-like *var* (CIDRα1.1)	**PFD0020c**
dbla_cidra	dc8-2	[16]	Two group B, DC8-like *var* (DBLα2_CIDRα1)	MAL6P1.316, PF08_0140
dblb12 & dblb3/5	dc8-3	[16]	DC8-like *var* in group A and B (DBLβ12, DBLβ3)	MAL6P1.316, PF08_0140, **PFD0020c, PF13_0003, PF11_0521 PFD1235w**
dblg4/6	dc8-4	[16]	DC8-like *var* in group A and B (DBLγ4, DBLγ6)	**PFD0020c,** MAL6P1.316, PF08_0140
B1	b1	[38]	Conserved upstream of group B *var*	
C1	c1	[38]	Conserved upstream of group C *var*	
Seryl-tRNA_synthatase		[39]		
Fructose_biphosphase aldolase		[39]		

“Primer name” is the name of the primer in the original study (see reference column), “Name given” is the name given to the primer in this study. We included the primers gpA3 and gpA4 designed based on 3D7 genome (a clone of NF54) to independently capture group A expression. Primers were previously shown to amplify the 3D7 genes shown in the right-hand column [16]. Gene names in bold are group A *var* genes. New 3D7 gene names: PFD0020c = PF3D7_0400400; PFA0015c = PF3D7_0100300; MAL6P1.314 = PF3D7_0600400; PFI1820w = PF3D7_0937600; PFD1235w = PF3D7_0425800; PFE1640w = PF3D7_0533100; PF11_0008 = PF3D7_1100200; PF08_0141 = PF3D7_0800200; PF11_0521 = PF3D7_1150400; PF13_0003 = PF3D7_1300300; MAL6P1.316 = PF3D7_0600200; PF08_0140 = PF3D7_0800300

Domain cassettes present a challenge for amplification by qPCR because they are defined as common combinations of domains whereas individual qPCR amplified domains can occur in different molecular contexts. We used primers designed to amplify DC8-like genes from field isolates [16]. Here we define DC8-like genes as those that contain individual PCR-targeted, DBL or CIDR domain sequence features present within the originally defined domain cassette 8 [7]. Specific data for amplification from the NF54-derived, 3D7 genome (Table 1) shows that dc8-1 amplifies a single group A gene PFD0020c containing a DC8-like CIDRa1.1 domain, dc8-2 amplifies two DC8-like group B genes that contain a characteristic DBLα2 domain joined to a CIDRα1 domain, dc8-3 and dc8-4 amplify DBLβ and DBLγ domains that are found in DC8, but in 3D7 are found in both group A and group B genes (Table 1).

The Real-time quantitative PCR was carried out in duplicates in 96-well plates. The PCR reaction and cycling conditions were carried out as described in [16] using the Applied Biosystems 7500 Real-time PCR system with a cycle threshold (Ct) set at 0.025 [40, 41]. Controls with no template were included and the melt-curves analysed for non-specific amplification. The ∆∆ct relative quantification method was used to calculate the arbitrary transcript unit (TU_s_) using the formula (TU_s_ = 2^(5-∆∆ct)^). Relative quantification is inappropriate for estimating proportional expression. Therefore, when calculating the proportional expression of the *var* subclasses, we used TU_s_ calculated from the formula (TU_s_ = 2^(5-∆ct)^) as described in [40, 41]. We assigned a TU_s_ value of zero to any reaction that did not give a detectable amplification after 40 cycles of amplification. Only samples where we could obtain amplification with the two housekeeping genes were included in the analysis.

### Infected erythrocyte surface antibodies (αIE)

Plasma obtained from study participants before CHMI was used to assess antibodies specific to IE (αIE) with the trophozoite stages of 8 clinical isolates recently adapted into culture [42] following isolation from children living in Kilifi county, Kenya. In vitro, the NF54 parasite line predominantly expresses var2CSA [43, 44] and from our experience, this line and other long-term laboratory cultured parasite lines give poor signal when reacted with plasma from individuals with naturally acquired immunity as compared with ex vivo matured clinical isolates. For that reason, we chose recently culture-adapted Kenyan isolates [42] to increase the breadth of variant surface antigens available for recognition by naturally acquired antibodies present in the plasma of the volunteers. The 8 isolates were mixed together in equal proportion based on parasitemia to make a composite of approximately 1.5% parasitemia before reacting with the plasma from each participant. A single plasma sample from an exposed adult residing in Kilifi, Kenya was used as a positive control and four from malaria-naïve Europeans plus one-pooled European sera of AB blood group were used as negative controls. The assay was carried out in duplicate and in two 96-well plates, providing four data points for each participant. Reactivity of plasma against the IE was measured using flow cytometry [10, 11] and presented as mean of the median fluorescent intensity obtained from two plates.

### Anti-schizont extract and anti-MSP2 antibodies

These data were obtained using ELISA as previously published [29].

### Statistical analysis

Statistical analysis was performed using Stata version 13 and graphs were generated using GraphPad Prism version 5.

When more than one primer was used to quantify a certain *var* subclass, we calculated the median transcript quantity obtained with the different primers to represent the expression of the particular *var* subclass as described in [40, 41]. For example, the median of DC8 (dc8_median) = the median transcript obtained with primers dc8-1, dc8-2, dc8-3, and dc8-4, and the median of group A (gpA_median) = the median transcript obtained with the primers gpA1, gpA3, and gpA4. The primer to DC13, specifically designed to target CIDRα1.4 was not included in this global calculation because it is known to amplify only a single *var* gene, PF11_0521 (Table 1).

Correlation between variables was assessed using Spearman’s rank correlation coefficient and Bonferroni correction was performed to adjust the *p*-value for multiple comparison (unless otherwise stated in the text, or indicted with * in the tables, quoted *p*-values are unadjusted). Linear regression analysis was used to assess whether the relationship between PMR and the transcript quantity of the *var* subclasses expressed by the infecting parasites was confounded by pre-existing antibodies to IE (αIE). To normalize the distribution of the data before use in regression analysis, the square root of the explanatory variables was calculated except for 1) group A proportional expression (gpA_prop and gpA_prop2) that was already normally distributed and 2) the αIE where the inverse (1/αIE) was taken.

Participant 110 from the published study [29] was excluded from analyses that includes *var* gene expression data, since amplification of the reference genes was not achieved due to the low parasitaemia.

## Results

### Pre-existing naturally acquired αIE differentially selects against group A and DC8-like *var* gene expression

To determine whether αIE antibodies carried by each volunteer before CHMI, imposed a selection pressure on the PfEMP1 antigens expressed in parasites that escaped those antibodies and established blood infection, we tested for correlations between αIE antibodies and expression levels of *var* gene subclasses encoding PfEMP1 associated with low antigenic diversity. We predicted that the parasites that establish blood-stage infection in volunteers with low naturally acquired pre-existing αIE antibodies would express higher levels of the conserved *var* gene subclasses, group A and DC8-like compared to those with high pre-existing αIE antibodies.

The results matched well with these predictions. The transcript quantity obtained with one of the primers designed to target the majority of group A *var* genes [16] (gpA1) was negatively associated with αIE (Table 2). The associations between gpA1 and αIE persisted after Bonferroni correction for ten comparisons (corrected *p* = 0.006, Table 2). The transcript quantity of two other primers (gpA3 and gpA4) also designed to target group A *var* genes and used in previously published studies [38, 45] showed a trend towards a negative association with αIE but with borderline significance (Table 2). The median transcript quantity of group A *var* genes (gpA_median) as measured with the three primers gpA1, gpA3, and gpA4 designed to globally amplify group A *var* genes was negatively associated with αIE (Table 2). However, DC13 primers, designed to amplify a subset of group A *var* genes containing CIDRa1.4 domains showed no evidence for an association with αIE antibodies.

**Table 2:** The relationship between expression of specific *var* subclasses, IE surface antibodies (αIE) and parasite multiplication rate (PMR)

	αIE		PMR		Parasitemia	
	*rho*	*p*	*rho*	*p*	*rho*	*p*
Transcript quantity (individual primers)						
gpA1	−0.62	0.0006*	0.44	0.02	0.08	0.7
gpA3	−0.38	0.05	0.38	0.049	0.14	0.5
gpA4	−0.37	0.05	−0.13	0.5	−0.22	0.3
dc13	0.07	0.7	−0.08	0.7	−0.03	0.9
dc8-1	−0.39	0.045	0.60	0.001*	0.18	0.4
dc8-2	−0.53	0.004*	0.43	0.03	0.13	0.5
dc8-3	−0.53	0.005*	0.13	0.5	−0.24	0.22
dc8-4	−0.56	0.003*	0.22	0.3	0.14	0.5
b1	−0.03	0.9	0.17	0.4	0.45	0.02
c1	−0.12	0.6	−0.34	0.08	−0.07	0.7
Transcript quantity (medians)						
gpA_median	−0.50	0.008	0.39	0.045	0.06	0.8
dc8_median	−0.57	0.002	0.44	0.02	0.05	0.8
Proportional expression						
gpA_prop	−0.70	0.0001*	0.52	0.006*	−0.04	0.9
dc8_prop	−0.47	0.01	0.42	0.03	−0.08	0.7
dc13_prop	0.11	0.6	−0.11	0.6	−0.05	0.8
b1_prop	0.31	0.1	−0.06	0.8	0.38	0.05
c1_prop	0.25	0.2	−0.51	0.006*	−0.15	0.4
Non-overlapping proportional expression						
gpA_prop2	−0.63	0.0005*	0.50	0.007*	−0.14	0.5
b1_prop2	0.33	0.1	−0.02	0.91	0.36	0.06
c1_prop2	0.28	0.2	−0.50	0.008*	−0.15	0.4

Shown is the Spearman’s correlation coefficient and uncorrected *p-value*. The names of the primers listed in Table 1 were used to represent the *var* subclasses. *Indicate *p*-value that was significant after Bonferroni correction for multiple comparisons (10, 5 and 3 comparisons for the transcript quantity, proportional expression and non-overlapping proportional expression respectively). *PMR* parasite multiplication rate, *αIE* antibodies to IE

The transcript quantity of the four primers targeting sequence features found within DC8 [16] dc8-1, dc8-2, dc8-3 and dc8-4 indicated negative trends in relation to αIE (Table 2). dc8-2, dc8-3, and dc8-4 transcript quantity scores were significantly negatively associated with αIE after Bonferroni correction for ten comparisons (corrected *p-value* = 0.04, 0.05, and 0.03 for dc8-2, dc8-3, and dc8-4 respectively, Table 2). The median transcript quantity of the four DC8 targeting primers (dc8_median) was also negatively associated with αIE (Table 2).

Group B and group C (as measured with the primers b1 and c1 respectively) showed no evidence for an association with αIE (Table 2).

In the above analyses, transcript quantities of the *var* subclasses were calculated relative to the average expression of two metabolic genes. This approach does not give information on the proportion contributed by each *var* subclass to the overall *var* expression of the parasite population causing the infection. To refine the analysis and better describe the relative ability of parasites expressing different PfEMP1 types to survive within infecting parasite population, we therefore estimated the expression of each of the broad classes of the *var* genes as proportion of the total measured *var* transcript as previously described [40, 41] (see methods) and assessed the relationship between the “proportional expression” values of each of the *var* subclass and αIE.

Proportional expression of Group A (gpA_prop) and DC8 (dc8_prop) showed a significant negative association with αIE (Table 2) but only gpA_prop remained significant after adjusting for five comparisons (adjusted *p* = 0.0005, Table 2). The associations between group B and C proportional expression (b1_prop and c1_prop), and αIE tended to be positive though not significant (Table 2).

Since different defined subgroups of *var* genes frequently carry shared sequence features, the transcript obtained with primer sets used to quantify the different *var* subclasses may overlap [40]. For examples, DC8 primers such as dc8-1 can amplify genes amplified by both the group A primers and primer b1 while the DC13 primer may amplify genes targeted by the group A primers since DC13 are a subset of group A. To estimate proportional expression of non-overlapping *var* classes, we excluded DC8 & DC13 from the calculation of total transcript and recalculated the proportions. With this analysis group A proportional expression (gpA_prop2) was negatively associated with αIE (Table 2) and remained significant after correcting for three comparisons (corrected *p* = 0.03).

### *var* expression patterns exhibit differential associations with apparent within-host parasite multiplication rate

One of the reasons proposed to explain why group A and DC8 PfEMP1 are relatively conserved is that they are adapted to mediate high levels of cytoadhesion [19, 21, 22, 46] potentially giving parasites expressing these variants a growth advantage, by lowering the rate at which they are cleared from the circulation by the spleen. We therefore tested the relationship between the transcript quantity of the *var* subclasses and PMR using Spearman’s rank correlation coefficient. *Var* transcript quantities measured using individual primer sets amplifying group A (gpA1, gpA3) and DC8 (dc8-1, dc8-2) showed a positive trend in relation to PMR (Table 2). However, only dc8-1 remained significantly associated with PMR after Bonferroni correction for 10 comparisons (corrected *p* = 0.01, Table 2). In contrast, no significant correlation was seen between the transcript quantities of dc13, group B (b1) and group C (c1) and PMR (Table 2). The median transcript quantity of group A (gpA_median) and DC8 (dc8_median) were also weakly associated with PMR (Table 2).

As a secondary analysis, we again re-calculated these associations using proportional expression measures. Like the transcript quantity, group A and DC8 proportional expressions (gpA_prop and dc8_prop) were positively associated with PMR (Table 2). In contrast, the proportional expression of group C (c1_prop) was negatively associated with PMR (Table 2). The proportional expression of DC13 and group B (dc13_prop and b1_prop) were not associated with PMR (Table 2). The association of both gpA_prop and c1_prop with PMR remained significant after adjusting for five comparisons (adjusted *p* = 0.03 for both gpA_prop and c1_prop, Table 2).

Similarly, after adjusting for possible overlap between primers in this calculation, PMR was positively and negatively associated with gpA_prop2 and c1_prop2 respectively (Table 2). These associations remained significant after correcting for three comparisons (adjusted *p* = 0.035 & 0.04 for gpA_prop2 and c1_prop2 respectively, Table 2). b1_prop2 showed no evidence for an association with PMR (Table 2).

To exclude the possibility that the association between expression of the different *var* subclasses and PMR was an artefact of the parasitemia at the time of sampling, we tested the relationship between the *var* expression and sampling parasitemia (p/mL). Only the transcript quantity of group B, which was not associated with PMR, showed a significant association with parasitemia at the time of sampling (Table 2).

### Antibodies to antigens on the surface of IE (αIE) provided an immunological correlate of low PMR

If antibodies to αIE play a direct role in the control of parasite multiplication by decreasing parasite survival, we would expect αIE to provide the best immunological correlate of low PMR. We therefore made a comparison of our measurement of αIE antibodies with previously published data available on the volunteers [29]. In the original study, participants were classified before CHMI into those with definite (DefExp) and minimal (MinExp) prior exposure to *P. falciparum* based on antibody levels to schizonts extract and MSP-2 [29]. All participants were diagnosed with malaria by blood-film apart from one DefExp participant (110) who was blood-film negative throughout follow-up. 18S qPCR targeting ribosomal RNA (18S rRNA) gene confirmed this subject was successfully infected with malaria parasites [29]. This volunteer had a reduced PMR (1.3) in comparison to the other twenty-seven volunteers (median PMR = 11.1) [29].

**Fig. 1:**
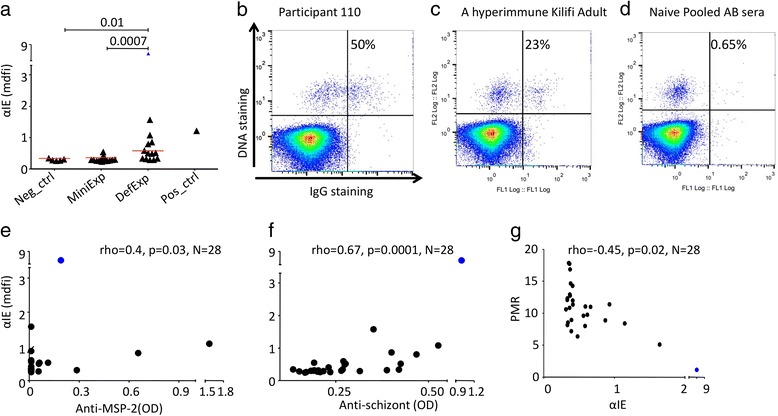
Naturally acquired pre-challenge IE surface antibodies (αIE). **a** Dot plot showing the αIE levels in relation to prior exposure to *P. falciparum* (Mann-Whitney U test). The red bar indicates the median level. **b**-**d** shows IE surface staining of IgG from participant 110 (**b**), a hyperimmune Kilifi adult (**c**), pooled naïve European sera (**d**). The upper right quadrant shows the percentage of IE recognized by the antibodies. **e**-**f** αIE association with anti-MSP-2 (**e**) and anti-schizont extract (**f**). **g** Relationship between PMR and αIE, *p* value was calculated using Spearman’s correlation coefficient. MinExp = minimal prior exposure, DefExp = Definite prior exposure, Neg ctrl = negative control, post ctrl = positive control. αIE = antibodies to IE, OD = optical density, MSP-2 = merozoite surface protein 2, AB = sera from AB blood group, mdfi = median fluorescent intensity. αIE level for participant 110 is highlighted in blue

Analysis of levels of αIE prior to CHMI showed a significant difference between the DefExp and MinExp groups (*p* = 0.0007, Fig. 1a, Mann-Whitney U test). The differences between the two groups hold even after excluding participant 110 (*p* = 0.001). Participant 110 reacted to the highest proportion of IE, surpassing the positive control (50%, 23% and 0.65% for participant 110, the positive and negative controls respectively, Fig. 1b-d)). αIE positively correlated with antibody levels to both MSP-2 and schizont extract (anti-MSP-2; rho = 0.4, *p* = 0.03, anti-schizont; rho = 0.67, *p* = 0.0001, Fig. 1e-f).

In these volunteers, antibodies to schizonts extract were negatively associated with the PMR [29] and no associations were observed with antibodies against MSP-2 [29]. We therefore tested the relationship between the αIE present before CHMI and the PMR. Consistent with the anti-schizont antibodies, the αIE was negatively associated with the PMR (rho = −0.45, *p* = 0.01, *N* = 28, Fig. 1g). When we excluded participant 110 who had very high αIE, this association remained significant (rho = 0.4, *p* = 0.04, *N* = 27). To test how αIE compares to anti-schizonts extract in predicting PMR, we used two linear regression models predicting PMR using either of them as an explanatory variable. As expected, both antibodies predicted PMR ((coeff(95%CI), anti-schizont = 1.0(0.16, 1.84), *p* = 0.02; αIE = 2.60(1.20, 4.0), *p* = 0.001). However, αIE explained 33% of the variations in PMR (adjusted R^2^ = 33%) while anti-schizont antibodies explained only 15.6% (adjusted R^2^ = 15.6%), suggesting αIE may be a better immunological correlate of PMR**.** Results from linear regression were consistent with those obtained using non-parametric Spearman correlation analysis (associations with PMR excluding volunteer 110: MSP-2, rho = −0.13, *p* = 0.53; anti-schizont, −0.31, *p* = 0.11; αIE, rho = −0.39, *p* = 0.04).

### Expression of a gene containing a CIDRα1.1 is associated with PMR independently of pre-challenge αIE antibody levels

Given that 1) both αIE and expression of specific subclasses of *var* genes were associated with PMR (Fig. 1g and Table 2) and 2) the expression of the subclasses of *var* genes were associated with the αIE (Table 2), we sought to dissect this network of relationships further to gain insight into possible causal pathways. Specifically, we tested for evidence that specific *var* classes were independently associated with higher PMR. We again used linear regression models predicting PMR. All variables whose association with PMR reached an unadjusted *p*<0.05 level of significance using Spearman’s rank correlation test (Table 2 and Fig. 1g) were used as explanatory variables. These explanatory variables were considered in turn in 11 separate models predicting PMR.

To determine the level of independence of αIE and *var* expression in predicting PMR, we considered each of the *var* gene expression estimates in combination with αIE**.** As shown in Table 3 models 1-11, consistent with the Spearman correlation analysis, each of the variables was significantly associated with PMR when used as the sole explanatory variable. Overall, the results suggest that the associations between expression of group A and DC8 *var* subclasses and PMR is confounded by the level of circulating αIE antibodies, as the expression of group A and DC8 no longer remained significantly associated with PMR after correction for prior levels of these antibodies (group A: models 12, 13, 15, 17, 20, DC8: models 16 and 18, Table 3).

**Table 3:** The relationship between αIE antibodies before CHMI, expression of specific *var* subclasses after CHMI and PMR (outcome measure). uncorrected *p*-values are shown

Models	Explanatory variables	Coeff(95% CI)	*p*-value	Adjusted R^2^
1	gpA1	1.18(0.06, 2.30)	0.04	13%
2	gpA3	1.55(0.06,3.05)	0.04	12%
3	dc8-1	1.73(0.62,2.85)	0.004	26%
4	gpA_median	0.33(0.03, 0.63)	0.03	14%
5	Dc8_median	1.32(0.07,2.58)	0.04	12%
6	gpA_prop	7.88(2.73,13.02)	0.004	26%
7	dc8_prop	7.85(−0.09,15.79)	0.05	11%
8	c1_prop	−9.26(−14.85, −3.66)	0.003	29%
9	gpA_prop2	7.25(2.77,11.73)	0.003	28%
10	c1_prop2	−8.93(−14.50, −3.37)	0.003	28%
11	αIE	1.99(0.42, 3.55)	0.02	18%
12	gpA1	0.42(−1.12,1.97)	0.6	16%
	αIE	1.55(−0.69,3.80)	0.17	
13	gpA3	0.91(−0.69,2.52)	0.3	20%
	αIE	1.54(−0.21,3.28)	0.08	
14	dc8-1	1.32(0.06,2.58)	0.04	29%
	αIE	1.12(−0.56,2.80)	0.2	
15	gpA_median	0.18 (−0.18, 0.53)	0.3	19%
	αIE	1.44 (−0.46, 3.35)	0.1	
16	dc8_median	0.66(−0.81, 2.14)	0.4	18%
	αIE	1.50 (−0.40, 3.41)	0.1	
17	gpA_prop	6.0 (−1.08, 13.10)	0.1	25%
	αIE	0.80 (−1.26, 2.85)	0.4	
18	dc8_prop	4.65(−3.67, 12.96)	0.3	19%
	αIE	1.59 (−0.12, 3.31)	0.07	
19	c1_prop	−7.31 (−13.45, −1.17)	0.02	32%
	αIE	1.13 (−0.47, 2.73)	0.2	
20	gpA_prop2	5.80(−0.32,11.92)	0.06	26%
	αIE	0.71(−1.30,2.72)	0.5	
21	c1_prop2	−7.0(−13.03, −0.95)	0.03	31%
	αIE	1.18(−0.43,2.78)	0.1	

However, 1) the expression of DC8 *var* amplified with the primer dc8-1, and 2) the measure of group C proportional expression, showed independent associations with PMR after adjusting for αIE, positively in the case of dc8-1 and negatively in the case of group C (Table 3 model 14, 19 and 21). Primer dc8-1 is known to amplify a single gene PFD0020c in the NF54-derived parasite line 3D7 [16], and expression of this gene was not strongly associated with αIE antibodies. This may suggest that this gene is capable of both evading host antibodies in this group of volunteers and promoting parasite survival.

## Discussion

We used controlled human malaria infection of Kenyan volunteers with varying levels of naturally acquired immunity to malaria to explore how parasites adapt to host antibodies during infections, through switching between alternative copies of PfEMP1 antigens inserted into the surface of parasite infected erythrocytes.

The aim of this study was to examine the inter-relationships between antibodies to parasite infected erythrocytes (αIE) carried by volunteers before experimental infection, the apparent within host multiplication rate of parasites during the subsequent infection (PMR) and the expression of specific sub-classes of parasite *var* genes in the surviving parasite population at the time before the infections were drug-treated. We focused on the expression of subsets of *var* genes previously shown to be associated with low host immunity and severe malaria. Taken together, the results show, for the first time, in a longitudinal study that parasite group A and DC8-like *var* expression is negatively associated with levels of αIE carried before challenge.

PfEMP1 stimulates an antibody response whose breadth develops progressively with increasing exposure to natural *P. falciparum* infections [5, 28]. To this effect, adults that grow up in malaria-endemic regions can control parasitemia and acquire protection against clinical malaria [47, 48]. Sera from these adults recognize many clinical isolates [49]. This ability to control parasitemia is thought to occur partly by preventing IE cytoadhesion and sequestration in the organs, making them more susceptible to removal by the spleen [50–54].

**Fig. 2:**
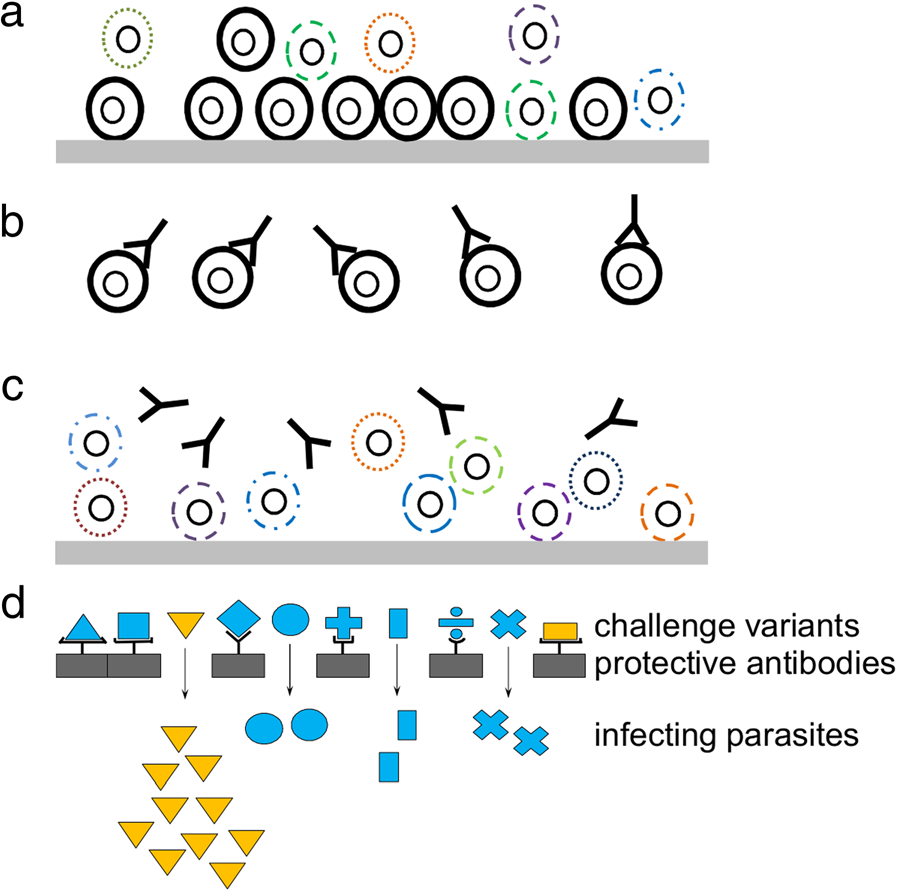
Proposed model to explain the inter-relationships between antibodies, *var* expression and apparent parasite multiplication rate (PMR). **a** In the absence of αIE antibodies, parasites expressing a subset of “dominant” PfEMP1 with high intrinsic cytoadhesive capacity dominate the infection (black) because of their ability to limit splenic parasite clearance rate. **b** As a result, these variants will be the first to be recognized by the developing host antibody response. **c** The surviving parasites express PfEMP1 variants that can evade antibodies, but because they have a lower intrinsic cytoadhesive capacity, these parasites have a higher splenic clearance rate, resulting in a lower observed apparent PMR. The thick grey horizontal line represents the endothelial cells that make up the inner wall of microvessels. **d** under a model of variant specific immunity, dominant variants (orange shapes) may arise that are poorly recognised by antibodies carried by the host population (grey rectangles) allowing them to establish infections (arrows). Their expression levels would be correlated with growth rate but poorly correlated with bulk measures of pre-infection antibodies

Earlier serological studies emphasized the importance of gaps in the pre-infection repertoire of protective antibodies to specific antigenic variants in explaining individual instances of clinical malaria [55, 56]. This subsequently developed into a model of immunity in which immune responses to a subset of dominant variants conferring enhanced parasite survival (group A and DC8) potentially explain the relatively rapid development of immunity to severe malaria relative to non-severe malaria or asymptomatic infection (Fig. 2a-c). The negative association between the naturally acquired αIE and expression of group A, DC8 and PMR is consistent with a role for these antibodies against in vivo expansion of parasite population (parasite burden).

If these two general ideas are combined, we arrive at a model in which variant specific antibodies control individual members of a subset of dominant variants. Under this modified model of variant specific immunity to dominant PfEMP1, we would expect to see examples of dominant PfEMP1 that are associated with PMR, but poorly associated with prior exposure, because of low herd immunity in the host population (Fig. 2d). The single group A gene PFD0020c, predicted to be amplified by the dc8-1 primers may provide an example of such a molecule. Unless the PFD0020c identified in the NF54-derived line 3D7 has undergone substantial rearrangement relative to the parent NF54 line used here, the results suggest that expression of a PFD0020c-like gene was significantly associated with PMR without a significant association with prior αIE antibodies to local isolates. In support of this, PFD0020c from the 3D7 line was notable in previous studies. 1) unlike other group A PfEMP1, PFD0020c was poorly recognized by pooled semi immune serum [26]; 2) in controlled infection of naïve volunteers, this gene was dominantly expressed [44]. It is perhaps also significant to note that the dblβ12 from this PFD0020c was recently found to bind to gCq1R [57] raising the possibly of an immune modulatory effect that might conceivably reduce antibody mediated clearance. Further studies are clearly needed to quantify the expression of individual *var* genes in relation to domain specific antibodies against recombinant PFD0020c domains in comparison with other PfEMP1 variants from NF54 and other local parasite isolates.

This study was limited to using primers with broad specificity and relatively crude assays to assess antibody carriage before parasite challenge. However, the results demonstrate the strong potential for the exposed volunteer CHMI platform for making a detailed dissection of the host parasite interaction during the development of naturally acquired immunity. Recent studies suggest that parasites may use a similar bet-hedging strategy seen in the *var* genes to adapt to changes in their host environment [58]. Several other parasite multi-gene families (including *rif* and *stevor* encoding exported parasite antigens and others encoding proteins with diverse functions, 6-cys, clag, etramp, acs, fikk and phist a,b and c) are expressed in a clonally variant manner. Future studies using a combination of protein arrays to make fine grained measures of pre-challenge antibodies and parasite RNAseq to assess how infecting parasite populations collectively respond to antibody pressure may now provide direct insight into how parasites establish and maintain infections.

## Conclusions

In summary, the results show that 1) naturally acquired αIE antibodies carried before controlled infection with NF54 strain PfSPZ appear to protect preferentially against Group A, and DC8-like *var* gene expression 2) expression of a *var* gene predicted to encode a DC8-like PfEMP1 similar to 3D7 PFD0020c, was associated with higher PMR after CHMI. We propose that, in non-immune individuals, absence or low αIE antibodies contributes to a reduction in parasite clearance resulting in increased apparent parasite multiplication rate (PMR). Within such a model, interventions targeting specific subsets of PfEMP1 may reduce parasite growth in vivo which may in turn reduce malaria associated mortality and morbidity.

## Abbreviations

CHMI: Controlled human malaria infection
DC13: Domain cassette 13
DC8: Domain cassette 8
IE: Infected erythrocytes
PfEMP1: *P. falciparum* erythrocyte membrane protein 1
PfSPZ: *P. falciparum* Sporozoite
PMR: Parasite multiplication rate
αIE: antibodies to IE
